# Amniotic Epithelial Stem Cells Counteract Acidic Degradation By-Products of Electrospun PLGA Scaffold by Improving Their Immunomodulatory Profile In Vitro

**DOI:** 10.3390/cells10113221

**Published:** 2021-11-18

**Authors:** Mohammad El Khatib, Valentina Russo, Giuseppe Prencipe, Annunziata Mauro, Ralf Wyrwa, Gabriele Grimm, Miriam Di Mattia, Paolo Berardinelli, Matthias Schnabelrauch, Barbara Barboni

**Affiliations:** 1 Unit of Basic and Applied Biosciences, Faculty of Bioscience and Agro-Food and Environmental Technology, University of Teramo, 64100 Teramo, Italy; melkhatib@unite.it (M.E.K.); vrusso@unite.it (V.R.); amauro@unite.it (A.M.); mdimattia@unite.it (M.D.M.); pberardinelli@unite.it (P.B.); bbarboni@unite.it (B.B.); 2 Department of Biomaterials, INNOVENT e.V., 07745 Jena, Germany; rw1@innovent-jena.de (R.W.); gg1@innovent-jena.de (G.G.); ms@innovent-jena.de (M.S.)

**Keywords:** PLGA scaffolds, electrospinning, tendon tissue engineering, ECM, biodegradability, hydrolytic degradation, amniotic epithelial stem cells, cytotoxicity, anti-inflammatory, degradation by-products

## Abstract

Electrospun poly(lactic-*co*-glycolic acid) (PLGA) scaffolds with highly aligned fibers (ha-PLGA) represent promising materials in the field of tendon tissue engineering (TE) due to their characteristics in mimicking fibrous extracellular matrix (ECM) of tendon native tissue. Among these properties, scaffold biodegradability must be controlled allowing its replacement by a neo-formed native tendon tissue in a controlled manner. In this study, ha-PLGA were subjected to hydrolytic degradation up to 20 weeks, under di-H_2_O and PBS conditions according to ISO 10993-13:2010. These were then characterized for their physical, morphological, and mechanical features. In vitro cytotoxicity tests were conducted on ovine amniotic epithelial stem cells (oAECs), up to 7 days, to assess the effect of non-buffered and buffered PLGA by-products at different concentrations on cell viability and their stimuli on oAECs’ immunomodulatory properties. The ha-PLGA scaffolds degraded slowly as evidenced by a slight decrease in mass loss (14%) and average molecular weight (35%), with estimated degradation half-time of about 40 weeks under di-H_2_O. The ultrastructure morphology of the scaffolds showed no significant fiber degradation even after 20 weeks, but alteration of fiber alignment was already evident at week 1. Moreover, mechanical properties decreased throughout the degradation times under wet as well as dry PBS conditions. The influence of acid degradation media on oAECs was dose-dependent, with a considerable effect at 7 days’ culture point. This effect was notably reduced by using buffered media. To a certain level, cells were able to compensate the generated inflammation-like microenvironment by upregulating IL-10 gene expression and favoring an anti-inflammatory rather than pro-inflammatory response. These in vitro results are essential to better understand the degradation behavior of ha-PLGA in vivo and the effect of their degradation by-products on affecting cell performance. Indeed, buffering the degradation milieu could represent a promising strategy to balance scaffold degradation. These findings give good hope with reference to the in vivo condition characterized by physiological buffering systems.

## 1. Introduction

A scaffold implanted in the body should provide sufficient mechanical properties and structural integrity to support the loads applied during the early stage of reparation until the engineered cells or autologous cells, attracted by the neighboring tissues, are able to synthesize sufficient ECM [1,2]. Moreover, biodegradability (bioresorbability) represents a key criterium that a scaffold must possess after accomplishment of its mission [1,2], so that its by-products are totally eliminated through natural metabolic pathways in the human body with no residual side effects [3].

Different polymers including poly(ϵ-caprolactone) (PCL), poly(lactic acid) (PLA), and poly(lactide-*co*-glycolide) (PLGA) are FDA-approved polymers that have been widely applied for biomedical and in particular tendon applications [1,2,4,5,6,7,8,9,10,11,12,13,14]. Although PLA and PCL are two biocompatible polymers, they are characterized by high hydrophobic properties that decreased cell adhesion due to the lack of cell recognition sites on their surfaces [9,15,16]. This might lead to a long in vivo degradation rate, which restricts their target applications depending on the regeneration time of the damaged tissue [9,15,16]. In particular, PCL has a long degradation time in vivo (around 2 to 4 years) and its poor cellular adhesion properties imply its use in combination with other materials to improve its bioactivity [16]. In addition, PLA is a crystalline polymer that degrades in vivo in degradable fragments leading to considerable inflammatory response in the body [16]. To overcome these drawbacks, PLGA, a copolymer made up of polylactic (PLA) and polyglycolic (PGA) acids [4], has emerged as promising material for scaffold fabrication. It is eliminated from the body after being metabolized through the tricarboxylic acid cycle (Krebs cycle) as carbon dioxide and water [17]. However, according to Stratton et al. [16], the major drawback of PLGA is related to its highly acidic degradation by-products that might be very difficult for the body to metabolize rapidly at high concentrations. For this reason, the degradation rate together with the mechanical strength of PLGA can be modulated through the variation of PLA ratio within the copolymer [18,19]. It was reported that PLGA degradation rates vary from several weeks to several months depending on different factors: shape of scaffold, copolymer composition (ratio of LA to GA), block or random copolymer, and molecular weight, as well as the environmental conditions in which the degradation process is assessed (i.e., medium, temperature, and pH) [20,21,22,23,24,25,26]. The degradation of polyester materials, especially PLGA, follows chemical cleavage of the hydrolytically cleavable covalent ester bonds, leading to a decrease in the polymer molecular weight. This degradation step is followed by an autocatalyze process [27] facilitated by the carboxylic acid ends, leading to massive cleavage of the backbone covalent bonds and hence spontaneous loss of polymer integrity [17,23]. It could be hypothesized that when PLGA is degraded in vivo, the release of acidic by-products might decrease the pH of the interest body district. This in turn should stimulate the physiological buffering systems that could influence the degradation process of PLGA. Within the body, pH is regulated through the blood plasma, kidneys, and respiratory system, which play the major role of physiological buffering systems [28,29]. Plasma proteins, which mostly function within the cells, phosphate, bicarbonate, and carbonic acid buffers are examples of the buffer systems found in blood plasma [28,29].

Electrospun PLGA scaffolds represent promising materials to be applied in the field of tissue engineering (TE) due to their aptitude in mimicking fibrous ECM of native tissues. They can be produced with determinate architectures in terms of fiber alignment and diameter depending on the tissue to be applied to [2,30,31]. Due to their versatility, PLGA scaffolds have been broadly utilized as temporary ECM for bone [32,33,34], tendon [35,36,37,38,39,40,41,42], nerve [43,44], salivary gland [45], and skin [46,47] regeneration.

In previous studies, it was verified that tendon biomimetic electrospun PLGA scaffolds with highly aligned fibers and adequate fiber diameter size (ha-PLGA) possess mechanical properties that are similar to human tendons [10,13,14]. Moreover, it was demonstrated that the developed constructs are suitable for inducing the differentiation of ovine amniotic epithelial stem cells (oAECs) towards the tenogenic lineage and for boosting their immunomodulatory properties by enhancing anti-inflammatory interleukin production [10,14], crucial for tendon regeneration.

Another critical factor for ECM formation and replacement is the optimization of scaffold degradation rate. Scaffolds that possess fast degradation rates might exhibit instantaneous mechanical destabilization and might provoke an increased release of acidic degradation by-products within the implantation site, hence altering the regeneration process [48]. Instead, scaffolds that degrade slowly might promote transport around the scaffolds for the whole regeneration period, in turn, fostering the formation of homogenized tissue, hence avoiding the alteration of cell function [49].

The evaluation of the degradation behavior of the tendon biomimetic electrospun PLGA scaffolds with highly aligned fibers has not been conducted yet. Instead, for tendon TE, degradation is considered a critical parameter to be monitored. In fact, it is important to understand the structural changes that occur to the scaffold during degradation, which in turn, may affect its mechanical properties and may also become cytotoxic, due to released by-products, for the cells engineered within the scaffolds or recruited at the implanted site [48,50].

Considering these premises, in this study, the in vitro degradation of PLGA scaffolds with highly aligned fibers (ha-PLGA), fabricated through a validated and standardized protocol via electrospinning [10,13,14], was investigated. The degradation experiments were conducted under ultrapure water (di-H_2_O) and phosphate buffer saline solution (PBS) conditions at 37 °C for long-term culture up to 20 weeks, according to Gil-Castel [23] and ISO 10993-13:2010, to simulate physiological conditions, since tendons usually have a very slow healing rate (up to 12 months) [51]. The PLGA scaffolds were characterized for their physical, morphological, and mechanical characteristics during degradation. Additionally, in vitro cytotoxic tests were conducted on oAECs to assess the effect of non-buffered and buffered PLGA by-products (LA, GA, and LA:GA) at different concentrations and to evaluate their stimuli on oAECs’ immunomodulatory properties.

## 2. Materials and Methods

### 2.1. Materials

The poly(_L_-lactide-*co*-glycolide) (PLGA; PLG8523) was provided by Corbion PURAC (Gorinchem, The Netherlands). It consisted of 85% _L_-lactide and 15% glycolide monomer units, with a molecular weight of M_w_ = 258,000 g.mol^−1^ measured using gel permeation chromatography (GPC) after dissolving the polymer in chloroform, where the polystyrene was as an external standard. Hexafluoro-2-propanol (HFIP) was obtained from Apollo Scientific (Manchester, UK) with a purity of 99%. All other chemicals and solvents were of analytical grade and used as received.

### 2.2. Synthesis of Poly(_L_-lactide-co-glycolide) Scaffolds

The fabrication of aligned PLGA scaffolds was performed by means of a commercial E-Spintronic electrospinning apparatus, developed at Erich Huber (Gerlinden, Germany). Briefly, 12% (*w/w*) PLGA solution was prepared by dissolving PLGA granules in pure HFIP under gentle stirring overnight at room temperature (RT). The polymer solution was fed into a 3 mL plastic syringe connected by 25 cm PTFE tube (Intra Special Catheters, Rehlingen-Siersburg, Germany) to a stainless-steel straight-end hollow needle (0.8 mm) under optimal conditions and electrospun at a 33 kV voltage, with a tip-to-collector distance of 20 cm and a solution flow rate of 0.25 mL.h^−1^. The temperature and the humidity of the electrospinning chamber were adjusted to 22.5 °C and 65%, respectively. Aluminum foil, placed on the metallic rotator collector drum of 12 cm diameter, set at 1000 rpm to obtain aligned microfibers, was used to collect the scaffolds. Each PLGA scaffold was obtained by electrospinning 500 µL of the polymer solution resulting in scaffolds of about 37 cm in length and 3 cm in width.

### 2.3. In Vitro Degradation

The electrospun ha-PLGA scaffolds were subjected to hydrolytic degradation under di-H_2_O and PBS, according to the international norm ISO 10993-13:2010, method 4.3. Shortly, the ha-PLGA scaffolds were cut into rectangular pieces (80 mm × 20 mm) with a mass of around 20 mg. The specimens were weighed (m_0_) and placed in a previously weighed vial (m_vial_). A volume of 10 mL of degradation medium was introduced, then the vials were sealed with parafilm and placed in a controlled incubator at 37 °C. At least three replicates of the hydrolytic degradation were considered for each assessment test at each time point for up to 20 weeks, as specified in each paragraph. Every week, half of the degradation medium was withdrawn and renewed by fresh medium [23].

### 2.4. Characterization of the Degradation Media: pH and Conductivity Measurements

The pH and conductivity of the degradation media were measured at RT at each time point using a pH meter and conductivity meter (WTW 82362, LF 330, Weilheim, Germany), respectively. Every week, from week 1 to week 20, the half-discarded degradation medium from each sample was collected and measured as described above. The pH meter was calibrated using three different buffer solutions: pH 4, pH 7, and pH 10.

### 2.5. Characterization of the Fabricated Poly(_L_-lactide-co-glycolide) Scaffolds during Degradation

#### 2.5.1. Wettability and Mass Loss Changes

The electrospun PLGA scaffolds were taken out from the PBS and di-H_2_O medium in time intervals (0, 1, 2, 4, 6, 8, 10, 12, 16, and 20 weeks). The scaffolds were gently dabbed to remove excess liquid and weighed to obtain their wet mass (*m_w_*). Afterward, these scaffolds were air dried overnight and weighed again to obtain their dry mass (*m_d_*). *The liquid uptake* of the scaffold, whether PBS or di-H_2_O, was calculated by Equation (1) below:

(1) $$\%\;liquid\;uptake=\left(\frac{m_{w}-m_{d}}{m_{d}}\right)$$

The initial mass of the scaffold (*m*_0_) was measured before it was immersed in the degradation medium (PBS and di-H_2_O), and the *mass loss* of the scaffold after degradation evaluation was measured, then calculated using the Equation (2):

(2) $$\%\;mass\;loss=\left(\frac{m_{0}-m_{d}}{m_{0}}\right)$$

#### 2.5.2. Gel Permeation Chromatography (GPC)

Molecular weights (M_n_ and M_w_) were determined by GPC using polystyrene (PSS-Polymer Standards Service, Mainz, Germany) as an analytical standard. The measurements were performed on a SECcurity^2^ GPC System (Agilent, Waldbronn, Germany). All samples were analyzed at RT. Chloroform (Fisher Scientific, Schwerte, Germany), stabilized with 1% amylene) was used as eluent, delivered at a flow rate of 1 mL.min^−1^. The samples were dissolved in chloroform at a concentration of 5 mg.mL^−1^. The injection volume was 50 µL. A pre-column PSS-SDV (100 Å, 8 × 50 mm) and columns PSS-SDV (1000 Å, 8 × 300 mm), PSS-SDV (100,000 Å, 8 × 300 mm), and PSS-SDV (1,000,000 Å, 8 × 300 mm) were used. A refractive index detector (1260 Infinity II RID, Agilent, Waldbronn, Germany) was used. Electrospun PLGA scaffolds under degradation were assessed for molecular weight changes at 0, 1, 4, 6, 8, 10, 12, 16, and 20 weeks.

#### 2.5.3. Scanning Electron Microscopy (SEM)

The surface morphology of the ha-PLGA scaffolds before and after each degradation time point was analyzed as follows. A Supra 55 VP field-emission scanning electron microscope (Carl Zeiss, Jena, Germany) at 5 kV accelerating voltage was used to evaluate the microfibers’ structure of the PLGA scaffolds after being sputtered with Au to ensure sufficient electric conductivity. ImageJ software (NIH) was used to calculate the average fiber diameter size and pore size using at least 100 fibers, randomly chosen for each sample, as well as the angle distribution of the electrospun fibers using the Directionality plugin (*n* = 3 for each sample/time point). This plugin chops the image into square pieces and computes their Fourier power spectra, allowing the generation of statistics on the basis of the highest peak found, represented by the center of the Gaussian (direction), the standard deviation of the Gaussian (dispersion), and the goodness of the fit, where 1 is good, and 0 is bad [13,52]. The ha-PLGA scaffolds subjected to degradation were analyzed at 0, 1, 10, and 20 weeks.

### 2.6. Mechanical Characterization

The electrospun ha-PLGA scaffolds under degradation were assessed for their mechanical properties under wet and dry conditions with stress-strain analysis conducted at RT using a TA.XT2i Texture Analyzer (Stable Micro Systems, Godalming, UK) with 5 kg load cells. For the mechanical characterization, only the ha-PLGA scaffolds under PBS conditions were considered. For the dry condition analysis, the samples were taken off the media, dabbed to remove liquid excess, dried in air for 5 h, and assessed for their mechanical properties together with those left in PBS that were removed from the incubator and subjected immediately to the mechanical test. The degraded samples were fixed with two clamps of the tester, then the test was started at a stretch speed of 1 mm.min^−1^ until the sample tore. The thickness of each sample was measured using a digital micrometer to calculate the cross-sectional area. Five samples for each time point (0, 1, 4, 6, 8, 10, 12, 16, and 20 weeks) were assessed. The obtained results are presented as elongation at break, ultimate tensile strength, and Young’s modulus by calculating the average results of five different measurements for each type of sample.

### 2.7. Ethical Statement

There is no need for any ethical declaration since the amniotic membranes used in this study were obtained from the discarded reproductive tissues of animals slaughtered at slaughterhouses.

### 2.8. Isolation of Ovine AECs

Amniotic membranes were collected from the discarded placentas of slain Appenninica breed sheep at roughly 2–3 months of pregnancy. Subsequently, ovine AECs (oAECs) were isolated from the obtained amniotic membranes of at least three fetuses, as described previously in Barboni et al. [53]. Briefly, oAECs were isolated from the epithelial layer of the amniotic membranes, after enzymatic digestion (0.25% Trypsin-EDTA, Sigma-Aldrich, St. Louis, MO, USA), previously washed with PBS, and cut aseptically into small pieces. Afterward, trypsin activity was inactivated by using fetal bovine serum (FBS) and the obtained cell suspension was filtered through a 40 µm cell filter into a 50 mL falcon tube. After centrifugation, cells were counted with vital trypan blue stain by using LUNA-II™ Automated Cell Counter (Logos Biosystems Inc., Gyeonggi-do, South Korea). Furthermore, oAECs were cultured in growth medium (GM) containing 20% FBS plus 1% penicillin-streptomycin, 1% amphotericin B, and 1% ultraglutamine at a concentration of 3 × 10^3^ cells/cm^2^ and incubated at 38°C with 5% CO_2_. Once cells reached 70% of confluence, they were detached using 0.25% Trypsin-EDTA, then plated in GM at the same cell concentration. Flow cytometry analysis was conducted to confirm the oAECs’ negative status for hemopoietic markers (CD14, CD58, CD31, and CD45), their positivity for surface adhesion molecules (CD29, CD49f, and CD166), stemness markers (TERT, SOX2, OCT4, and NANOG), a low expression for MHC class I molecules, the absence of MHC class II (HLA-DR) antigens [29,34,36], and their negativity for SCX, COL1, and TNMD gene expression [53].

### 2.9. Media Preparation and pH Measurements

Non-buffered and buffered (B) media containing lactic (LA), glycolic acid (GA), or a mixture of both LA:GA (1:1) were prepared by dissolving the acids at different concentrations (5, 10, 20, and 40 mM) directly in complete GM (20% FBS). For the buffered (B) media (LA-B, GA-B, and LA:GA-B), the pH was adjusted to 7.4 with concentrated NaOH solution. The media were then filtered using a 0.22 µm pore diameter filter and used immediately. Fresh culture media was prepared before each cell culture medium exchange.

The pH of the degradation media was measured at RT by Beckman device (Beckman Coulter, Inc., Brea, CA, USA). To calibrate the pH meter, three buffer solutions—phthalate buffer solution (pH 4.01), phosphate buffer solution (pH 7.00), and borate buffer solution (pH 10.00) (Certipur^®^, Sigma-Aldrich, Darmstadt, Germany)—were used. Measurements for each medium were carried out in triplicate, and the averages were taken as representative values (Table 1).

### 2.10. Cell Culture

The oAECs were cultured for MTT assay (2 × 10^4^ cells/well using 96-well plate) and for morphology and molecular PCR analysis (5 × 10^4^ cells/well using 24-well plate) in the presence of GM and incubated at 38 °C with 5% CO_2_ until reaching 70% of confluence. Then, cells were treated for 24 h, 48 h, and 7 d with non-buffered or buffered culture media of PLGA degradation by-products LA, GA, or mixture LA:GA, at concentrations of 5, 10, 20, and 40 mM. oAECs cultured in GM were used as control (CTR).

### 2.11. In Vitro Cytocompatibility

#### 2.11.1. MTT Assay

To evaluate the effects of the PLGA degradation by-products (non-buffered or buffered) on oAECs proliferation and viability, the MTT assay (M5655-1G, Sigma-Aldrich, St. Louis, MO, USA) was performed at 24 h, 48 h, and 7 d, according to the manufacturer’s instructions. Briefly, CTR and treated oAECs with acids, non-buffered and buffered, supplemented media (5, 10, 20, and 40 mM), were seeded into 96-well plates (2 × 10^4^ cells/well) until reaching 70% confluence. Wells without cells, containing only GM, were used as assay internal control (blank points). At the end of each culture period, 20 µL of MTT (5 mg/mL in PBS) was added to each well, and the plates were incubated for 3.5 h at 38.5 °C. After that, 100 µL of DMSO/each well was added to dissolve the formazan crystals. The absorbance (Abs) was measured at 595 nm by using EnSpire^®^ Multimode Plate Reader (PerkinElmer, Waltham, MA, USA). The percentage of viability was estimated according to Equation (3) [54]:

(3) $$\%\;Viability=\frac{Absorbance\;of\;treated\;cells}{Absorbance\;of\;CTR\;cells}\times 100$$

The net absorbance of *CTR* cells was assumed to represent 100% proliferation.

#### 2.11.2. Morphological Evaluation of the Cells

Microscopic analysis to evaluate the morphological changes in oAECs subjected to different PLGA degradation by-products treatments (5, 10, and 20 mM) were performed at the end of each time-culture period (24 h, 48 h, and 7 d) by fixing cells in 4% paraformaldehyde (15 min) at RT. After washing with PBS, cell morphology was evaluated by using a Nikon inverted microscope ECLIPSE Ti connected to a Nikon DS-Fi2 interfaced with a computer workstation loaded with NIS-Elements Advanced Research imaging software for the image captures.

#### 2.11.3. Reverse Transcription and Real-Time Polymerase Chain Reaction (RT-PCR)

The reverse transcriptase quantitative real-time polymerase chain reaction (RT-qPCR) method was used to compare immunomodulatory related genes in CTR and oAEC treated with non-buffered or buffered PLGA degradation by-products at different concentrations (5, 10, and 20 mM) for 24 h, 48 h, or 7 d. Total RNA was extracted using TRIzol (Sigma-Aldrich, St. Louis, MO, USA), according to previously published reports [10,14]. One µg of total RNA from each sample was utilized for cDNA synthesis using an RT reaction with Random Hexamer Primers and Tetro Reverse Transcriptase (Bioline, Germany) in a final volume of 20 µL. SensiFAST^TM^ SYBR Lo-ROX Kit (Bioline) was used to perform real-time qPCR analysis employing genes’ primer sequences for pro-inflammatory interleukin 12b (IL-12b) and anti-inflammatory interleukin 10 (IL-10) associated genes, according to the manufacturer’s instructions. The reaction was carried out with 7500 Fast Real-Time PCR System (Life Technologies, Waltham, MA, USA) using a two-step cycling protocol for 40 cycles (10 s at 95 °C for denaturation and 30 s at 60 °C for annealing/extension), followed by melt-profile analysis (7500 Software version 2.3). Each sample was run in triplicate for each gene expression, and the results were standardized to the endogenous GAPDH reference gene. The relative gene expression ratio (2^−∆∆Ct^) was computed using the comparative Ct (∆∆Ct) method.

### 2.12. Statistical Analysis

At least three replicates were used for each experiment at each time point, and the analyses concerning the biological experiments using oAECs were performed from at least three different fetuses (*n* = 3 biological replicates). The results in this study were expressed as mean ± standard deviation (SD). The results were assessed for normal distribution using D’Agostino–Pearson tests. Statistical analyses for assessing the fiber diameter size and the dispersion of fiber alignment, as well as those concerning the biological experiments, were performed using the one-way ANOVA multi-comparison tests followed by Tukey post hoc tests (GraphPad Prism 9, San Diego, CA, USA). The comparison between experimental conditions was performed with two-way ANOVA multi-comparison tests followed by Tukey post hoc tests (GraphPad Prism 9, San Diego, CA, USA).

## 3. Results

### 3.1. Changes of Ultrastructural Morphology after In Vitro Degradation on Electrospun Aligned PLGA Scaffold

**Figure 1:**
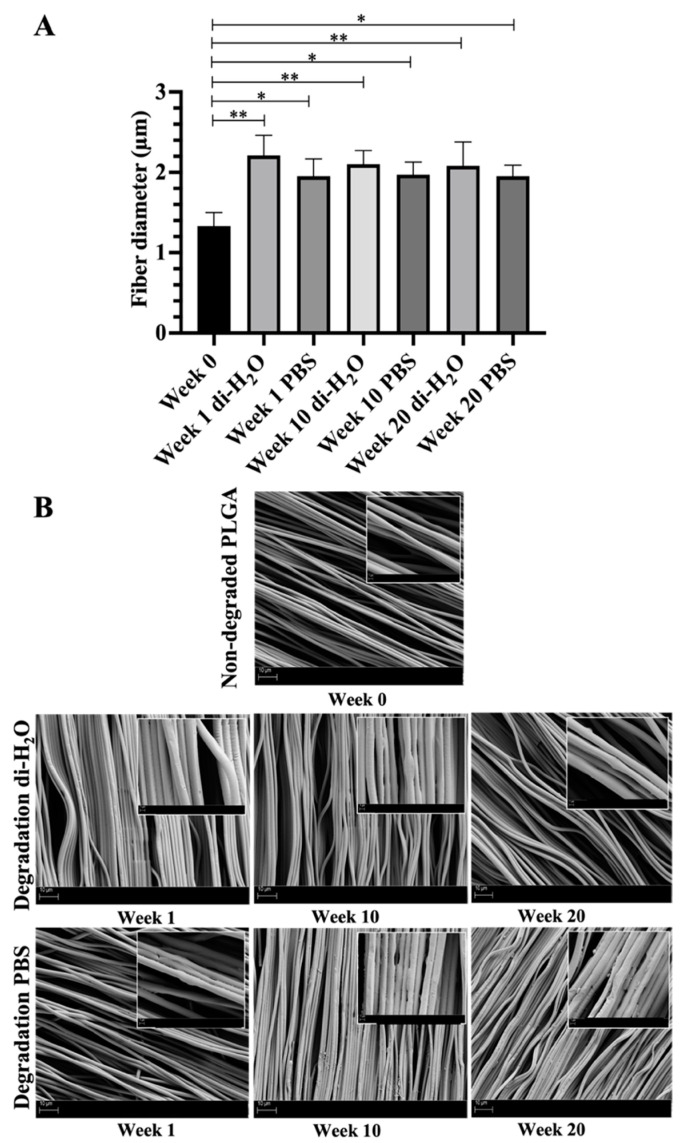
(**A**) Histogram showing the different fiber diameter sizes of the undegraded and degraded PLGA scaffolds at different time points. * and ** statistically between PLGA week 0 (non-degraded) and the PLGA scaffolds at different degradation time points (*p* < 0.05 and *p* < 0.01, respectively). (**B**) SEM investigations (5 kV, 10 µm) of the electrospun PLGA scaffold surface subjected to degradation under di-H_2_O and PBS at weeks 0, 1, 10, and 20. The inset in each image shows the same sample at higher magnification (5 kV, 1 µm).

The variation of fiber diameter size and surface morphology of the ha-PLGA scaffolds subjected to the in vitro hydrolytic degradation procedure was evaluated using SEM before and after 1, 10, and 20 weeks of degradation under di-H_2_O and PBS conditions (Figure 1).

The average fiber diameter size increased significantly after 1 week of degradation under both di-H_2_O and PBS conditions, with respect to PLGA at week 0, with higher values in the case of di-H_2_O (2.20 ± 0.25 and 1.95 ± 0.21 µm for di-H_2_O and PBS, respectively, vs. 1.33 ± 0.17 µm for PLGA; *p* < 0.05, Figure 1A). Along the degradation time, after 10 and 20 weeks of degradation under both di-H_2_O and PBS conditions, fibers exhibited similar values compared to fibers analyzed at week 1 (*p* > 0.05, Figure 1A). SEM images showed no significant fiber degradation (i.e., no pore formation within the fibers, no fiber ruptures) even over the 20 weeks. Instead, already from week 1, fiber alignment was affected throughout the experiments (Figure 1B).

**Figure 2:**
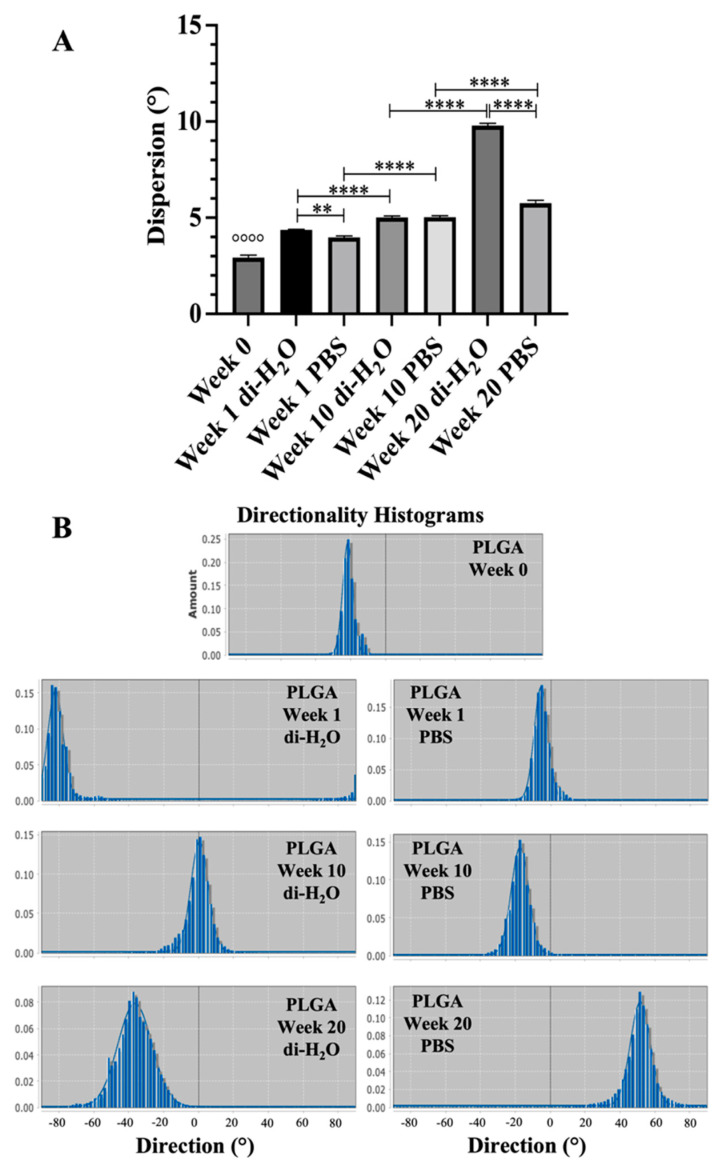
(**A**) The histogram shows the changes in fiber alignment of week 0 and electrospun PLGA scaffolds throughout 20 weeks of degradation under both di-H_2_O and PBS conditions. °°°° statistically significant between the different studied groups compared to PLGA week 0 (*p* < 0.0001). ** and **** statistically significant between the degraded PLGA scaffolds (*p* < 0.01 and *p* < 0.0001, respectively). (**B**) Representative histograms of each sample showing the angle distribution on the electrospun PLGA scaffolds throughout the degradation studies.

In particular, to assess the loss of fiber alignment, quantification experiments conducted. Before the degradation experiments at week 0, the fiber topology of the fabricated ha-PLGA scaffolds exhibited a high fiber alignment represented by a sharp Gaussian curve with a dispersion value of about 2.93 ± 0.12° (Figure 2). During degradation under both conditions and at all time points, the curves started to become broader, characterized by a significant increase in the dispersion angle of the Gaussian curves (Figure 2; *p* < 0.0001), indicating fiber alignment loss. In detail, after 1 week of degradation, fibers started to partly lose their alignment, and the curves representing the angle distribution of the fibers became broader (Figure 2), characterized by dispersion angles of around 4.63 ± 0.04° and 3.98 ± 0.08° compared to the non-degraded (week 0) PLGA scaffolds (*p* < 0.0001). The broadness of the curves increased with the degradation time (Figure 2) and the dispersion angles after 20 weeks of degradation reached values of about 9.80 ± 0.11° and 5.75 ± 0.15° under di-H_2_O and PBS conditions, respectively (*p* < 0.0001).

### 3.2. Weight Loss and Liquid Uptake of Electrospun PLGA Scaffolds

**Figure 3:**
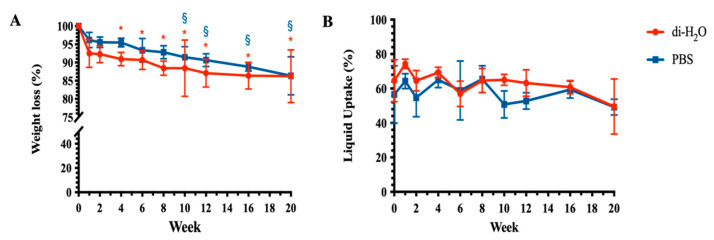
(**A**) Weight loss and (**B**) liquid uptake of electrospun PLGA scaffolds degraded in PBS and di-H_2_O solutions at 37 °C up to 20 weeks. * and ^§^ statistically significant vs. week 0 (*p* < 0.05). Statistical symbols in blue and red refer to di-H_2_O and PBS, respectively.

The ha-PLGA scaffolds showed a relatively slow hydrolytic degradation rate during the whole period of the in vitro experiments (Figure 3). In detail, the tested constructs after 1 week lost around 8% and 4% of their initial mass under di-H_2_O and PBS conditions, respectively, even if not significantly with respect to week 0 (*p* > 0.05). Instead, the PLGA constructs started to show a significant mass loss compared to week 0 starting from week 4 and week 10 of degradation under di-H_2_O and PBS conditions, respectively (*p* < 0.05 vs. week 0, Figure 3A). At the end of the degradation experiments, week 20, the ha-PLGA scaffolds under both degradation conditions displayed a similar weight loss characterized by approximately 14% (*p* < 0.0001 vs. week 0; Figure 3A). Although, this result demonstrates that these constructs exhibited a slow and not complete degradation profile.

The changes in liquid uptake property, under PBS and di-H_2_O, are shown in Figure 3B. Under both conditions, the liquid uptake exhibited a trend from increase to decrease even though never reaching significant values with respect to week 0. In detail, after 1 week of degradation, the liquid uptake increased from around 60% at week 0 to a maximum of 74 ± 3% for PBS and 64 ± 4% for di-H_2_O (*p* > 0.05), then decreased gradually until reaching its lowest value at the end of degradation studies, where at 20 weeks in both conditions, the liquid uptake showed similar values (49 ± 16% for PBS and 49 ± 5%, *p* > 0.05; Figure 3B).

### 3.3. pH and Conductivity Changes of the Degradation Media

**Figure 4:**
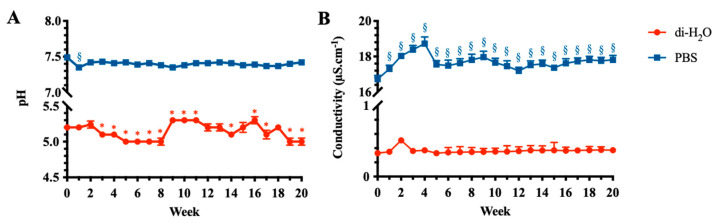
Degradation characteristics of the electrospun PLGA scaffolds under PBS and di-H_2_O conditions. (**A**) pH and (**B**) conductivity values of PBS and di-H_2_O along the degradation studies. * and ^§^ statistically significant vs. week 0 (*p* < 0.05). Statistical symbols in blue and red refer to di-H_2_O and PBS, respectively.

The variation of pH and conductivity values of PBS and di-H_2_O solutions during the degradation of the electrospun PLGA scaffolds are shown in Figure 4. In particular, the pH values of PBS decreased slightly after 1 week from 7.49 ± 0.03 to 7.35 ± 0.02 (*p* < 0.05) before returning nearly to its initial value of 7.43 ± 0.04 and remaining intact and unchanged throughout the whole degradation period (*p* > 0.05; Figure 4A). Under di-H_2_O conditions, pH decreased from 5.20 ± 0.03 at week 0 to reach 5.00 ± 0.05 (*p* < 0.05) until the end of the experiment (Figure 4A).

The evaluation of the liquid degradation fraction was assessed in terms of conductivity. When measured in di-H_2_O media, conductivity remained constant, showing values around 0.36 µS.cm^−1^ along the degradation period times (*p* > 0.05; Figure 4B). Instead, under PBS conditions, the conductivity increased significantly after the first week of degradation from 16.73 ± 0.20 µS.cm^−1^ to a maximum of 18.73 ± 0.37 µS.cm^−1^ (*p* < 0.05) at week 4, then decreased again from week 5 to week 20 to reach a value of around 17 µS.cm^−1^, significantly higher than compared to week 0 (*p* < 0.05; Figure 4B).

### 3.4. Change of Molecular Weight and Its Distribution of Electrospun PLGA Scaffolds Subjected to Degradation

**Figure 5:**
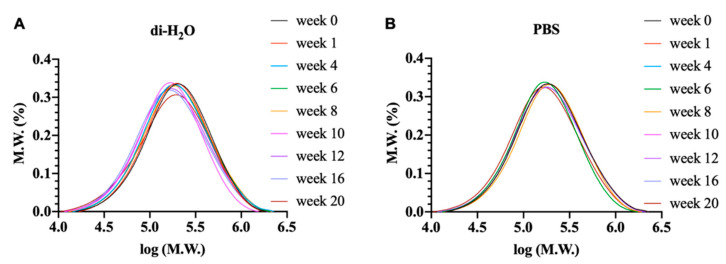
GPC curves of electrospun PLGA scaffolds degraded in (**A**) PBS and (**B**) di-H_2_O at 37 °C at marked degradation time.

The molecular weight distribution (MWD) of ha-PLGA scaffolds subjected to di-H_2_O and PBS media was also evaluated via GPC measurement (Figure 5). A unimodal distribution was observed for the non-exposed PLGA scaffold (week 0) that showed a sharp peak at 5.2 g.mol^−1^. When the electrospun scaffolds were immersed in di-H_2_O (Figure 5A), MWD exhibited a linear decrease from 143,631 g.mol^−1^ at week 0 to 94,570 ± 6345 g.mol^−1^ at 20 weeks (*p* < 0.0001). Under PBS conditions (Figure 5B), PLGA scaffolds experienced a slight exponential decrease reaching a value of 102,874 g.mol^−1^ at the end of week 20 (*p* < 0.0001). The MWD showed a slight shift to the left under both degradation conditions with a relatively more relevant profile under H_2_O conditions. The obtained MWD profile hypothesizes the hydrolytic degradation of the ester bonds with the subsequent chain scission within the PLGA scaffolds.

**Figure 6:**
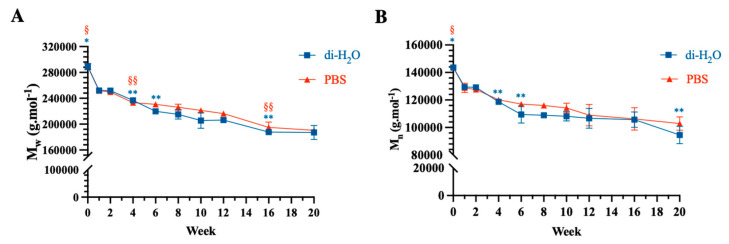
Changes of (**A**) number (M_n_) and (**B**) average molecular weight (M_w_) of electrospun PLGA scaffolds throughout degradation period under PBS and di-H_2_O conditions. * and ^§^ statistically significant vs. each degradation time point (*p* < 0.05). ** and ^§§^ statistically significant vs. consecutive degradation time points (*p* < 0.05). Statistical symbols in blue and red refer to di-H_2_O and PBS, respectively.

The number and weight average molecular weights (M_n_ and M_w_) of all ha-PLGA scaffolds degraded under both PBS and di-H_2_O conditions decreased throughout the degradation period (Figure 6). In detail, the average molecular weight (M_w_) of the tested ha-PLGA fell sharply after only 1 week of degradation, of about 12.78% and 12.85% under di-H_2_O and PBS conditions, respectively (*p* < 0.0001 vs. week 0). Afterward, the M_w_ decreased significantly and gradually until reaching 35.25% and 34.07% under di-H_2_O and PBS conditions, respectively, after 20 weeks of degradation (*p* < 0.01 vs. each degradation time point; Figure 6A).

Moreover, the number average molecular weight (M_n_) exhibited two sharp decreases along the degradation period, followed by a gradual one (Figure 6B). The first one was similar to M_w_, where M_n_ decreased sharply to about 11.62% (di-H_2_O) and 10.32% (PBS) after 1 week of degradation (*p* < 0.0001). The second decrease was between weeks 2 and 6, where M_n_ showed values of about 23.82% and 18.54% under di-H_2_O and PBS (*p* < 0.001). Subsequently, M_n_ continued to diminish gradually until attainment of 34.15% and 28.37% of loss under di-H_2_O and PBS, respectively, by the end of week 20 (*p* < 0.0001 vs. week 0; Figure 6B).

**Figure 7:**
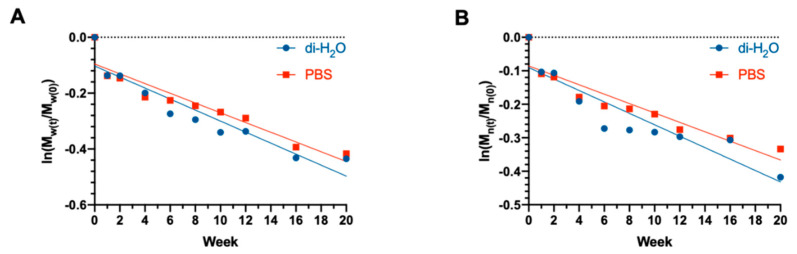
Semi logarithmic representation of the degradation of electrospun PLGA scaffolds up to 20 weeks according to (**A**) M_w_ and (**B**) M_n_.

According to Figure 7A,B, both M_n_ and M_w_ decreased exponentially throughout the degradation period. The apparent degradation rate (*K*) was calculated by assuming the following exponential relationship between molecular weight and degradation time according to Equation (4):

(4) $$\ln\frac{M_{t}}{M_{o}}=-K.t$$

where *K* is the apparent degradation rate, *M_t_* and *M*_0_ are the number or weight average molecular weight at each degradation time point, respectively, and *t* = 0, and *t* is the time represented by week^−1^. The degradation half-time $t_{1/2}$ can be further calculated using the following Equation (5):

(5) $$t_{1/2}=\frac{\ln\left(2\right)}{K}$$

The apparent degradation rates of ha-PLGA scaffolds based on M_n_ were 0.017 and 0.014 week^−1^ for di-H_2_O and PBS conditions, respectively, basically coherent with those based on M_w_ (0.020 and 0.017 week^−1^). The degradation half times calculated based on M_n_ for di-H_2_O and PBS conditions were about 40.77 and 49.51 weeks, respectively.

### 3.5. Change of Mechanical Properties of Electrospun PLGA Scaffolds during Degradation

**Figure 8:**
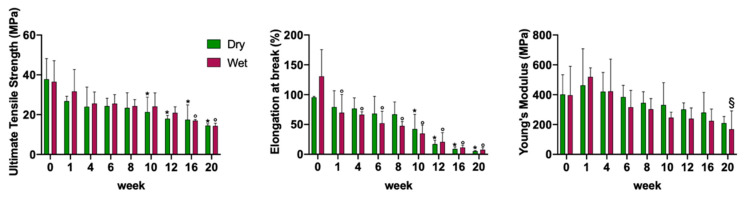
Mechanical properties of electrospun PLGA scaffolds in dry and wet conditions in PBS at different degradation time points. * statistically significant between non-degraded (week 0) and degraded PLGA scaffolds under dry conditions (*p* < 0.05). ^°^ statistically significant between non-degraded (week 0) and degraded PLGA scaffolds under wet conditions (*p* < 0.05). ^§^ statistically significant between PLGA scaffolds degraded at week 1 and week 20 under wet conditions (*p* < 0.05).

Mechanical properties were assessed in terms of ultimate tensile strength (UTS), elongation at break, and Young’s modulus of degrading ha-PLGA scaffolds at each degradation time point under both wet and dry conditions as described previously. The obtained results are illustrated in Figure 8.

The UTS property was held during degradation under both dry and wet conditions until weeks 8 and 12, respectively, compared to week 0 (*p* > 0.05). Afterward, the ha-PLGA scaffolds started to become fragile and lost their mechanical properties. In detail, UTS property started to decrease significantly under the dry conditions at week 10 until reaching its lowest value at the end of degradation by week 20 (14.5 ± 0.9 MPa for week 20 vs. 37.8 ± 10.4 MPa for week 0, *p* < 0.05). In contrast, for the wet electrospun ha-PLGA scaffolds, the UTS values decreases significantly later by week 16 compared to week 0 until reaching the lowest value by week 20 (14.3 ± 1.3 MPa; *p* < 0.05).

The same trend was observed by evaluating the elongation at break of the degrading electrospun PLGA scaffolds (Figure 8). The wet electrospun ha-PLGA scaffolds showed higher elongation at break value compared to dry conditions at week 0. The elongation at break decreased significantly and gradually starting from week 1 in wet conditions until reaching 7.8 ± 3.5% by week 20 compared to week 0 (130.8 ± 44.7%; *p* < 0.05). The gradual decrease of elongation at break under dry conditions started to be relevant and significant by week 10, and it continued to fall until attaining 5.4 ± 1% by week 20 compared to week 0 (95.5 ± 1.8%; *p* < 0.05).

In contrast, Young’s modulus property was not significantly affected by the degradation study, since it maintained a value around 348 MPa during the course of the study up to 20 weeks under both wet and dry conditions. It can be noticed that Young’s modulus values under dry and wet conditions increased slightly after 1 week (280.5 ± 135.1 and 423.6 ± 215.6 MPa for dry and wet conditions, respectively, *p* > 0.05) then decreased until reaching their lowest values by week 20 (209.9 ± 44.1 MPa for dry conditions vs. 1 week, *p* > 0.05 and 168.8 ± 122.9 MPa for wet conditions vs. 1 week, *p* < 0.05).

### 3.6. Effect of PLGA Degradation By-Products on oAECs

#### 3.6.1. MTT Assay on oAECs Treated with Non-Buffered and Buffered By-Products

oAECs viability study was performed at early (24 h and 48 h) and late (7 d) time points to obtain insights on the effect of PLGA degradation by-products.

**Figure 9:**
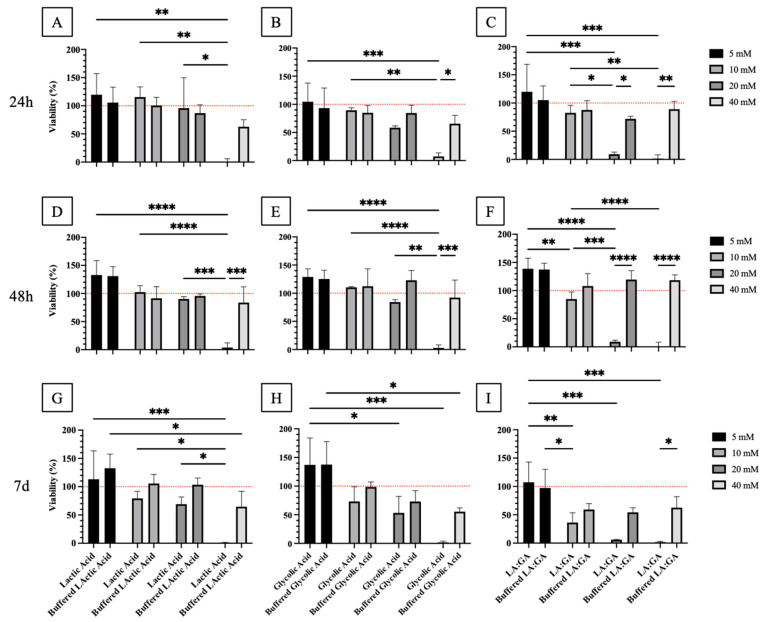
MTT oAECs analysis on the effect of PLGA degradation by-products (**A**,**D**,**G**). Lactic acid (LA) and buffered LA (**B**,**E**,**H**), glycolic acid (GA) and buffered GA, and (**C**,**F**,**I**) LA:GA and buffered LA:GA at different concentrations (5, 10, 20, and 40 mM) at 24 h, 48 h, and 7 d. CTR viability value (100%) has been used as reference (dotted red line). *, **, ***, and **** statistically significant between tested groups (*p* < 0.05, *p* < 0.01, *p* < 0.001, and *p* < 0.0001, respectively).

oAECs’ viability decreased proportionally with increasingly higher concentrations of non-buffered PLGA degradation by-products media (5, 10, 20, and 40 mM of LA, GA, or LA:GA) along with the culture time points (Figure 9). Alternatively, by buffering the degradation culture media, cell viability increased compared to cells treated with the non-buffered ones, and this was striking noticeable after 7 d culture (Figure 9). This result was in accordance with the morphology evaluation.

In particular, oAECs subjected to 40 mM LA non-buffered acidic media showed significantly lower vitality with respect to CTR and all other concentrations at the same time point (*p* < 0.05; Figure 9A,D,G). However, when buffered LA media were used at the same concentration, surprisingly the cell mortality was reversed and a significant difference between 40 mM LA and LA-B (*p* < 0.001) was observed (Figure 9A,D,G). Instead, at 7 d, a significant decrease in cell vitality was shown with 40 mM LA-B with respect to 5 mM LA-B (*p* < 0.05; Figure 9G).

The same correlation of results between acid concentration increase and viability decrease, observed after LA treatments, was also found in oAECs treated with GA. Treatment at 40 mM of GA was lethal for cells, and significant differences were found between 5 mM and 40 mM (*p* < 0.001) for all the time points (Figure 9B,E,H). In detail, a significant viability decrease was observed between 10 and 40 mM (*p* < 0.05) at 24 h (Figure 9B) and 48 h (Figure 9E), between 20 and 40 mM at 48 h (*p* < 0.05) (Figure 9E), and between 5 mM and 20 mM at 7 d (*p* < 0.05; Figure 9H). Otherwise, when buffered GA media were used, this correlation was minimized, and remarkably reversed cell death was observed with 40 mM GA. Then, significant differences were shown at 24 h and 48 h between 40 mM GA and 40 mM GA-B (*p* < 0.05; Figure 9B,E).

Interestingly and different from those observed with LA and GA treatments alone, when oAECs were treated with the mixture LA:GA, their viability was strongly negatively affected starting from LA:GA 20 mM. Indeed, non-buffered acidic media at 20 and 40 mM concentrations resulted lethal for all the time points (Figure 9C,F,I). A significant viability decrease was also observed in general between 5 mM and the other concentrations (*p* < 0.001; Figure 9C,F,I), depending on the time culture and more markedly compared to single acid treatments (Figure 9C,F,I). Also in this treatment, when buffered LA:GA media were used, vitality increased in particular at 20 and 40 mM LA:GA (*p* < 0.05) compared to non-buffered media at the same concentrations (Figure 9C,F,I).

Finally, based on the negative results observed on vitality at 40 mM, this concentration was excluded in the further experiments concerning the cellular morphology and gene expression evaluation.

#### 3.6.2. oAECs’ Morphology Evaluation after Treatment with Non-Buffered and Buffered By-Products

**Figure 10:**
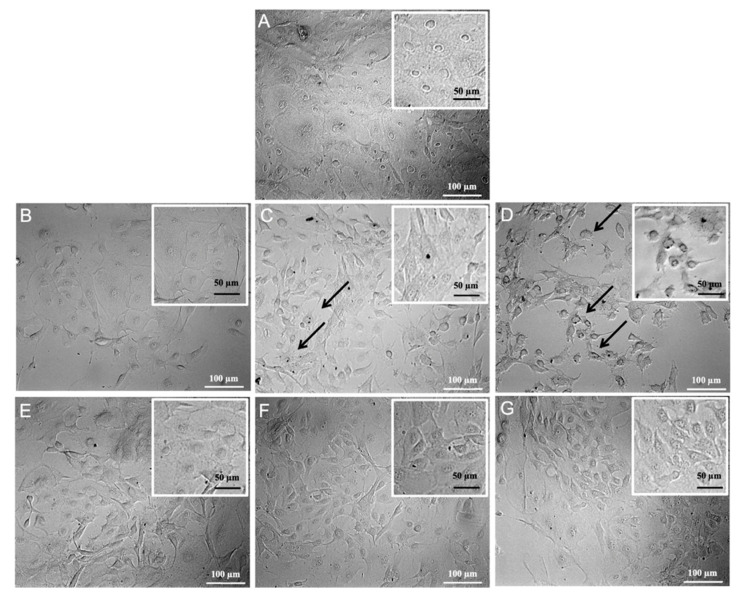
An example of phase contrast inverted microscope images of LA non-buffered and buffered treated oAECs. (**A**) The control cells (CTR) at 24 h were present in high density and showed a polygonal shape with regular dimension. (**B**) The cells exposed to 5 mM LA (24 h) did not differ from CTR 24 h. (**C**) The cells treated at 10 mM LA (24 h) started to show a suffering status (indicated by arrows). (**D**) The cells treated at 20 mM LA (24 h) became sparse, presenting pyknotic nuclei and blebs (indicated by arrows). (**E**) The cells exposed to 5 mM, (**F**) 10 mM, and (**G**) 20 mM buffered LA (24 h) did not differ from CTR 24 h. Scale bar of large images = 100 µm; scale bar of inset images = 50 µm.

oAECs appeared with a typical epithelial cobblestone shape in the control group (CTR; Figure 10A). Instead, after treatment with 5, 10, and 20 mM of LA, GA, or LA:GA (1:1) solutions, after 24 h, cells became sparse, presenting some pyknotic nuclei and blebs that increased at higher acid concentrations, suggesting suffering signs with respect to CTR 24 h (Figure 10B–D). Cellular suffering phenotype, under all conditions, was also observed in treated cells at 48 h and 7 d compared to their respective CTRs, which on the contrary, were characterized by a healthy cell appearance (data not shown). In contrast, cells treated with buffered media, even with increased acid concentrations, did not seem to suffer from the same condition of non-buffered, acid-treated cells (Figure 10E–G), suggesting a pH-dependent role in cell morphology changes.

#### 3.6.3. Immunomodulatory Response of oAECs

**Figure 11:**
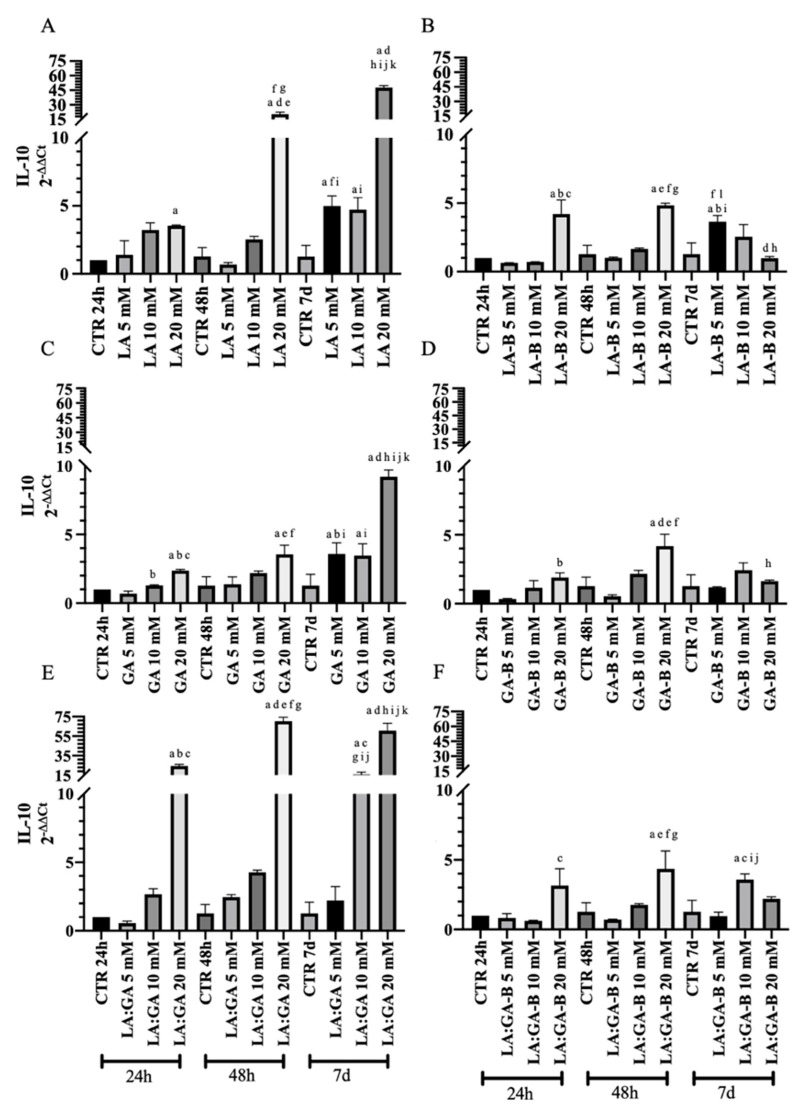
IL-10 expression after the treatments with non-buffered (**A**) LA, (**C**) GA, and (**E**) LA:GA and with the equivalents buffered (**B)** LA-B, (**D**) GA-B, and (**F**) LA:GA-B solutions at different concentrations and at different time points. Significant difference legend: ^a^ vs. CTR 24 h; ^b^ vs. 5 mM 24 h; ^c^ vs. 10 mM 24 h; ^d^ vs. 20 mM 24 h; ^e^ vs. CTR 48 h; ^f^ vs. 5 mM 48 h; ^g^ vs. 10 mM 48 h; ^h^ vs. 20 mM 48 h; ^i^ vs. CTR 7 d; ^j^ vs. 5 mM 7 d; ^k^ vs. 10 mM 7 d; and ^l^ vs. 20 mM 7 d.

**Figure 12:**
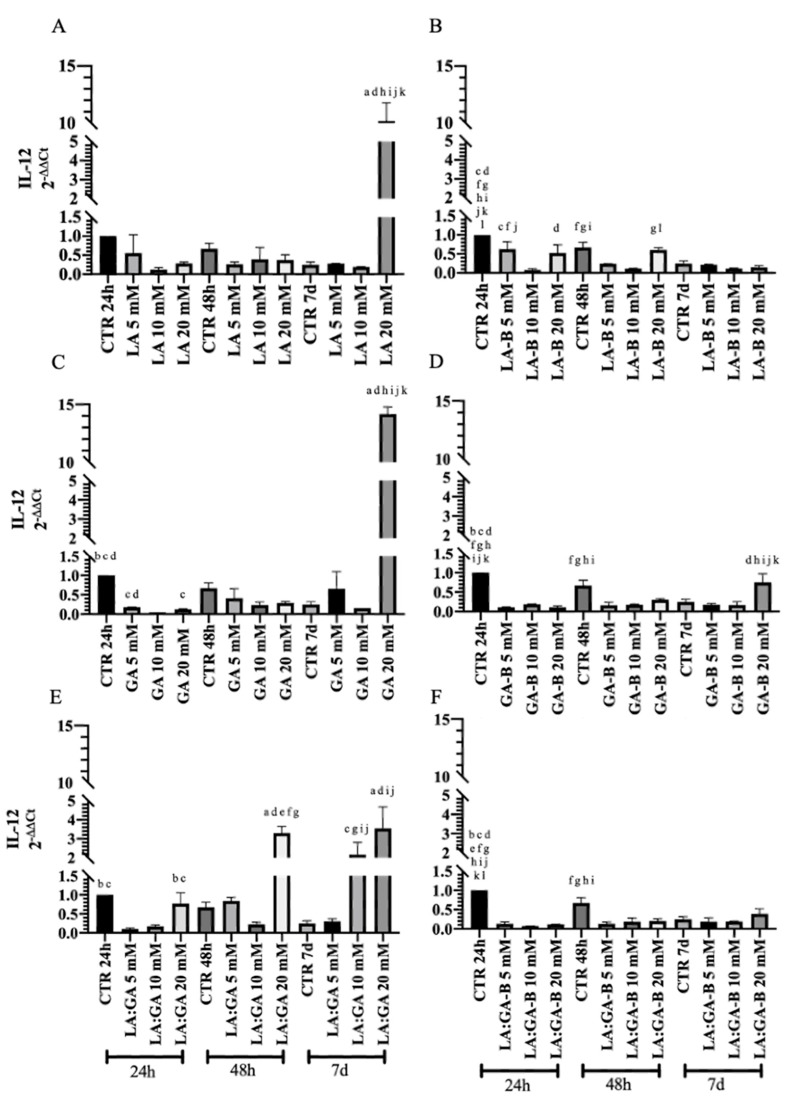
IL-12 expression after the treatments with non-buffered (**A**) LA, (**C**) GA, and (**E**) LA:GA and with the equivalents buffered (**B**) LA-B, (**D**) GA-B, and (**F**) LA:GA-B solutions at different concentrations and at different time points. Significant difference legend: ^a^ vs. CTR 24 h; ^b^ vs. 5 mM 24 h; ^c^ vs. 10 mM 24 h; ^d^ vs. 20 mM 24 h; ^e^ vs. CTR 48 h; ^f^ vs. 5 mM 48 h; ^g^ vs. 10 mM 48 h; ^h^ vs. 20 mM 48 h; ^i^ vs. CTR 7 d; ^j^ vs. 5 mM 7 d; ^k^ vs. 10 mM 7 d; and ^l^ vs. 20 mM 7 d.

The gene expression analysis of key anti-IL-10 (Figure 11) and pro-IL-12 (Figure 12) inflammatory cytokines was performed at early (24 h and 48 h) and late (7 d) stages of cell culture to obtain insights on the effect of PLGA degradation by-products (buffered or non-buffered) on the oAECs’ immunomodulatory properties. Cells cultured in GM for 24 h, 48 h, and 7 d were used as control (CTR). The expression profile of cytokines in oAECs was differently modulated, along with the culture time points, depending on cell exposure to PLGA degradation by-products (LA, GA, or LA:GA) at 5, 10, and 20 mM concentrations (buffered or non-buffered).

It was demonstrated that IL-10 mRNA expression upregulated preferentially with high PLGA degradation by-product concentrations and long culture time points. In fact, oAECs exposed to non-buffered degradation media, at the concentration of 20 mM, significantly upregulated IL-10 gene expression with respect to CTR (*p* < 0.05; Figure 11A,C,E). Moreover, at 7 d culture, oAECs subjected to 5 mM and 10 mM non-buffered degradation media exhibited significantly higher expression of IL-10 with respect to CTR 24 h and CTR 7 d (*p* < 0.05; Figure 11A,C,E), except for cells cultivated with 5 mM LA:GA mixture (*p* > 0.05; Figure 11A,C,E). However, this upregulation at 7 d culture remained lower than that of cells subjected to a 20 mM concentration (*p* < 0.0001; Figure 11A,C,E). Also, the above-discussed upregulation was notably reduced by buffering PLGA degradation by-product media at all considered concentrations (Figure 11B,D,F). However, a significant increase in the expression of anti-inflammatory cytokine IL-10 was observed with 20 mM at 48 h compared to the CTR and to other groups within the same culture time point (*p* < 0.05; Figure 11B,D,F).

The analysis of IL-12 revealed that oAECs exposed to PLGA non-buffered degradation by-products showed lower expression with respect to CTR 24 h. However, at 7 d culture, this trend was not upheld by exposing cells to 20 mM non-buffered acidic media, in which IL-12 expression was strongly upregulated with respect to CTR 24 h and other groups within the same culture points (*p* < 0.001; Figure 12A,C,E). Instead, when mixture LA:GA was used, upregulation of IL-12 was also noticed in the case of oAECs subjected to 20 mM at 48 h and 10 mM at 7 d with respect to CTR 24 h and other groups within the same culture time points (*p* < 0.05; Figure 12E). When oAECs were exposed to buffered PLGA degradation by-products media, a notable downregulation of IL-12 was observed, minimizing the toxic effects on oAECs subjected to high mixture acidic concentrations (Figure 12B,D,F).

**Figure 13:**
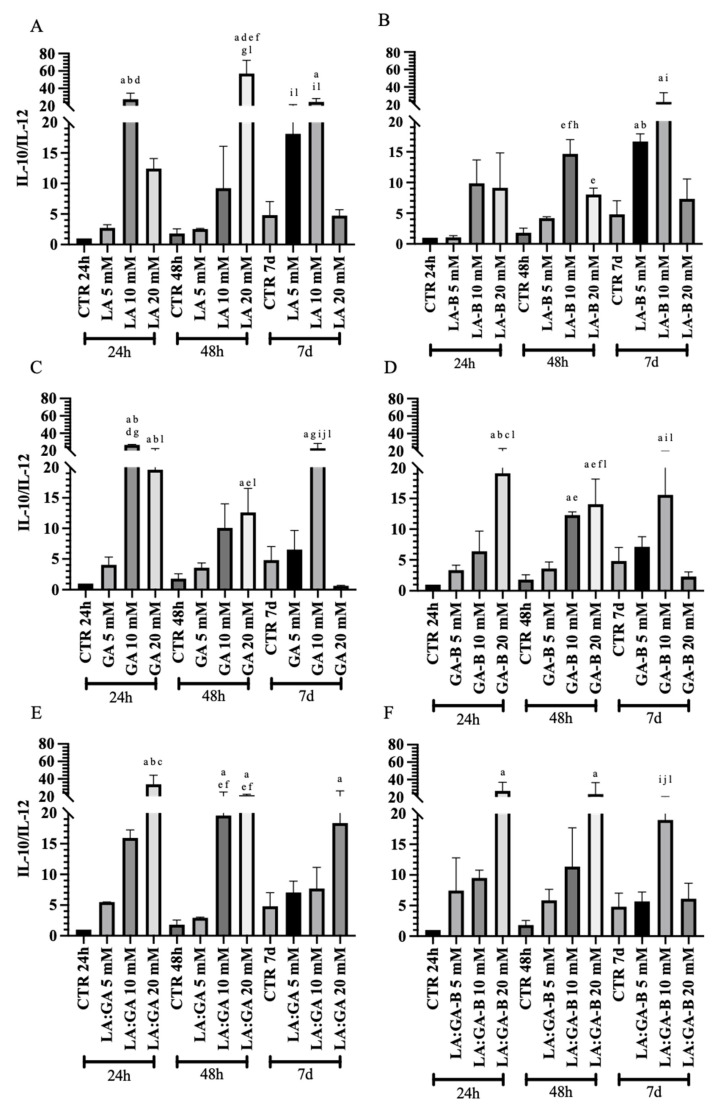
IL-10/IL-12 ratio of oAECs treated with non-buffered (**A**) LA, (**C**) GA, and (**E**) LA:GA and the equivalents buffered (**B**) LA-B, (**D**) GA-B, and (**F**) LA:GA-B solutions at different concentrations and at different time points. Significant difference legend: ^a^ vs. CTR 24 h; ^b^ vs. 5 mM 24 h; ^c^ vs. 10 mM 24 h; ^d^ vs. 20 mM 24 h; ^e^ vs. CTR 48 h; ^f^ vs. 5 mM 48 h; ^g^ vs. 10 mM 48 h; ^h^ vs. 20 mM 48 h; ^i^ vs. CTR 7 d; ^j^ vs. 5 mM 7 d; and ^l^ vs. 20 mM 7 d.

The analysis of IL-10/IL-12 ratio (Figure 13) indicated higher values in oAECs treated with PLGA non-buffered degradation by-products media at the different culture time points with respect to CTR 24 h. This trend arose noticeably by subjecting cells to 10 mM LA or GA media for 24 h and 7 d, which revealed an increased IL-10/IL-12 ratio with respect to CTR (*p* < 0.05; Figure 13A,C). The same profile was observed by subjecting cells to a 20 mM concentration for 24 h and 48 h (*p* < 0.05; Figure 13A,C,E), except for LA 20 mM at 24 h (*p* > 0.05; Figure 13A). Interestingly, oAECs showed a decreased IL-10/IL-12 ratio at 7 d culture when exposed to 20 mM degradation media with respect to the other concentrations within the same time point (Figure 13A,C), except for LA:GA 20 mM that exhibited a significantly higher value with respect to CTR (*p* < 0.05; Figure 13E). Moreover, by using buffered media, there was a favorable anti-inflammatory condition rather than a pro-inflammatory one (Figure 13B,D,F). In detail, at 24 h and 48 h culture, the cells subjected to a 20 mM concentration showed a similar profile characterized by a significant increase in IL-10/IL-12 ratios when subjected to GA-B with respect to CTR and other groups within the same time point (*p* < 0.05; Figure 13D) and to LA:GA-B only with respect to CTR (*p* < 0.05; Figure 13F). Furthermore, at 48 h and 7 d culture, a significant increase in IL-10/IL-12 ratio was noticed in the cells exposed to 10 mM LA-B, GA-B, and LA:GA-B with respect to CTR (*p* < 0.05; Figure 13B,D,F). This increase was only noted after 7 d culture in the cells exposed to 5 mM LA-B with respect to CTR (*p* < 0.05; Figure 13B).

## 4. Discussion

In TE, biodegradable synthetic polymers represent promising solutions due to their biocompatibility, non-risk of pathogen transmission, good mechanical properties, and large batch-to-batch scalability [55]. In TE, the neo-formation of ECM within the tissue has to be stimulated and modulated by the scaffold in terms of structure and morphology to match the targeted clinical application. Scaffold degradation also represents a critical feature, since it must be controlled during tissue remodeling to avoid tissue malformation, hence impacting tissue quality and regeneration [49].

For this reason, in this study for the first time to our knowledge, electrospun ha-PLGA scaffolds, designed for tendon TE, were subjected to hydrolytic degradation under di-H_2_O and PBS conditions at 37 °C and evaluated for up to 20 weeks. Of note, tendons regenerate at a slow healing rate, which necessitated performing the degradation study up to 20 weeks, compared to most other studies that usually conducted similar experiments on electrospun ha-PLGA constructs for no longer than 4 to 8 weeks [56,57,58,59,60,61,62]. The changes in scaffolds’ morphology, mass, liquid uptake, molecular weight, and distribution, as well as their mechanical properties, together with the changes in pH and conductivity, were investigated systematically. Moreover, the effect of PLGA degradation by-products (LA, GA, and LA:GA) was assessed in terms of cytotoxicity, by means of cellular morphology and MTT analysis, and of immunomodulation, by analyzing IL-10 and IL-12 gene expression profile, on oAECs.

PLGA copolymer is considered a hydrophobic biomaterial in biomedical applications due to the methyl groups present in the PLA side [63]. However, PLGA was shown to absorb water, leading consequently to its degradation by cleavage of ester covalent bonds [4]. In fact, ha-PLGA scaffolds showed a slow degradation rate represented by a loss of about 14% of their initial mass after 20 weeks with a faster rate in the case of di-H_2_O. The scaffolds showed an increase in the liquid uptake, represented by their swelling property, after week 1 of degradation, followed by a slight decrease maintained throughout the degradation period. This result was confirmed by the fact that no significant changes were noticed in pH and conductivity, since similar values to week 0 were obtained over the degradation periods. In a study conducted by Pamula et al. [25], they showed that PLGA films composed of 85% and 15% of PLA and PGA, respectively, exhibited no notable changes in pH and conductivity during the first 10 weeks in the ultrahigh quality water (UHQW) medium [25]. However, rapid decrease of pH and increase of conductivity of UHQW medium were noted at weeks 12 and 14. Even if the same PLGA composition was used in this study, the rapid decrease of pH and increase of conductivity after week 10 of degradation could be attributed to other factors, such as the solvent-casting technique used to fabricate PLGA films [25]. Probably this condition gave the scaffolds different characteristics that, in turn, accelerated the degradation rate compared to the electrospun ha-PLGA scaffolds used in this study. Notably, different degradation profiles were shown in previous publications reflecting the variability of PLGA biopolymer degradation based on many factors. For instance, some authors showed that electrospun PLGA scaffold lost 40% of its initial mass after 8 weeks [58], while no degradation was noted after 3 months in the case of PLGA films [25] and electrospun meshes [64] under PBS and serum conditions, respectively. These different results could be attributed to the enantiomeric isomers of PLGA itself, the ratio of LA to GA, its molecular weight, crystallinity, scaffold shape, and ultrastructure, as well as to the medium in which the degradation study was conducted.

When subjected to hydrolytic degradation, the ester bonds were cleaved, and the polymer chains were split expecting, in turn, a shift of the molecular weight to lower values. When exposed to both di-H_2_O and PBS conditions, MWD displaced slightly with a diminution of the M_w_ peaks’ height clearly evident under di-H_2_O. The M_w_ was found to decrease gradually over the entire degradation period. Since electrospun polymer scaffolds are porous in nature, in contrast to polymer blocks, the decrease in molecular weight was an essential change that may occur, influencing, in turn, the changes in other properties. The ha-PLGA scaffolds seemed to degrade faster under di-H_2_O due the fact that acidic compounds released from the polymer chains can be buffered by the PBS, thus, in turn, slightly decreasing the rate of degradation [23]. In fact, electrospun PLGA scaffolds under di-H_2_O conditions lost around 34% of their M_w_ compared to a 28% M_w_ loss under PBS. The obtained results were slightly different from those obtained by Chor et al., who demonstrated that electrospun PLGA (85:15) scaffolds with randomly oriented fibers lost around 50% of their M_w_ and M_n_ after 30 d of degradation in simulated body fluid [60]. The difference in molecular weight loss can be attributed to the variation in the M_w_ of the non-degraded electrospun PLGA scaffolds at week 0, the fiber topography, and the dimension of the PLGA scaffolds subjected to degradation. It can be hypothesized that the fiber alignment within the electrospun scaffolds might have also led to a better fluid exchange between the fibers, allowing acidic components to be quickly released from the degradation area due to diffusion and capillary forces that normally occur in a biological system, hence leading to the decrease of the scaffold degradation rate.

According to the degradation kinetics, it was possible to estimate the degradation half-life of the ha-PLGA scaffolds. It was found that these constructs exhibited a slow degradation profile represented by a degradation half-time of around 40 and 50 weeks under di-H_2_O and PBS conditions, respectively. This estimation could explain the obtained weight loss results confirming the long degradation capability of the constructs. This could be attributed to the high M_w_ of the polymer, as well as to the high LA ratio compared to GA, that decreased the degradation rate due to its hydrophobic property [65,66].

The above-obtained results were also confirmed by the SEM analyses conducted on the non-degraded and degraded ha-PLGA scaffolds. When incubation time was prolonged, the fiber diameter size of ha-PLGA scaffolds increased, indicating their swelling, due to the hydration phase, without observing any sign of degradation or partial deterioration of the bulk properties of the biomaterial. Similar trend profiles were observed for fiber diameter size and liquid uptake, where both parameters increased after 1 week of degradation to maintain a static trend throughout the degradation studies up to 20 weeks. It can be hypothesized that these findings are related to the ultrastructure of the electrospun ha-PLGA scaffolds with aligned fibers, which entrapped the air penetration, hence limiting the water penetration and subsequentially decreasing degradation [67]. Moreover, the degraded constructs gradually lost their fiber alignment over the degradation study due to the disruption of the internal fibers’ hydrogen bonds due to water penetration into the amorphous region of the scaffolds [17,23]. These results were in accordance with other studies that confirmed the long degradation rate of electrospun fibrous materials compared to films made of the same polymers [67]. This could be explained by the slow diffusion rate of degraded products containing acid-ending groups from the films inducing an autocatalytic effect in the center of the films, thus accelerating the degradation kinetic of the scaffold [67,68,69,70]. In contrast, the porous structure of the electrospun fibers rendered the diffusion of degraded oligomers easier, limiting such autocatalysis and slowing the degradation of the scaffold [23,68,71,72]. However, it was demonstrated that under cell culture conditions, the degradation profile of PLGA scaffolds might be facilitated and accelerated by the enzymes secreted by the cells [58,60,71,73].

For tendon TE, the scaffold should provide sufficient physical stability and mechanical properties for a certain period to support neo-tissue formation and regeneration of the damaged tissue. Over 20 weeks, the mechanical properties of ha-PLGA scaffolds showed to be influenced by the degradation time even if a significant decrease of the UTS was starting to be noticed by week 10. The decrease in UTS property could be related to the swelling effect due to the penetration of water into the polymer matrix. Indeed, by week 10, an increase was noticed in the dispersion parameter of fiber alignment that became higher by increasing degradation, explaining the changes in the UTS. Elongation at break seemed to be the parameter most influenced by the degradation, since it showed a significant gradual decrease throughout the degradation time that was more relevant under wet conditions as compared to dry ones. Moreover, Young’s modulus increased after 1 week of degradation before it decreased again until reaching the lowest value by week 20. These findings were in accordance with previous data that showed a decrease in the mechanical properties of the scaffolds subjected to degradation and analyzed under dry conditions [66,74]. It must be noted, though, that when scaffolds were implanted in vivo, the recruited cells at the implanted site started forming a sort of tissue-like ECM, which might, in turn, compensate for the lost mechanical properties of the biodegradable scaffolds, hence supporting tissue remodeling and its mechanical properties.

The effect of acidic degradation by-products at different concentrations of ha-PLGA scaffolds is of particular interest to assess cell cytotoxicity and biological response since the released products might affect the regeneration process by developing an inflammatory response around the implantation site.

Inflammation that can also be caused by the degradation by-products is a critical process that aims to remove pathogens, initiate the regeneration process, and contribute to restored homeostasis within the damaged tissues [75]. Inflammation represents a helpful defense reaction that may eliminate infections, mend wounded tissues, and restore damaged tissues and organs to their homeostasis state. An early self-limiting inflammation is critical for initiating a good healing process [75,76,77,78]. It was demonstrated that AECs exhibit remarkable regeneration capacity, which was linked to their ability to regulate inflammation [79]. In particular, these amniotic-derived cells play a role in modulating the complicated biological processes via established paracrine mechanisms that physiologically influence inflammation at the maternal–fetal interface and develop compensatory mechanisms that maintain this tolerance during pregnancy [80]. AECs have the ability to produce growth factors involved in tissue regeneration, such as hindering tissue fibrosis and immunosuppression of both constitutive and lipopolysaccharide (LPS)-induced immune systems [81,82,83].

oAECs are able to secrete cytokines and growth factors with antiapoptotic, proangiogenic, and immune-modulatory molecules, which can alter the pro-inflammatory signals and enhance the anti-inflammatory ones, hence improving tissue regeneration [84,85,86,87,88]. Among them, it was recently demonstrated in the amniotic epithelium that a highly conserved physiological mechanism amongst species controlling the expression and releasing balance of potent anti-inflammatory and proinflammatory cytokines, such as interleukin (IL)-10, IL-4, and IL-12, under both basal and LPS-inductive conditions [54,80,89,90].

To mimic an inflammation-like environment due the release of PLGA degradation by-products, oAECs were exposed to chemical insult by subjecting them to different concentrations of LA and GA. The selection of degradation by-product concentrations was based on previous studies conducted by Gil-Castel et al. [23] and Meyer et al. [91], where both worked with LA and GA concentrations in the range of 0 mM and 50 mM to assess their toxicity on fibroblasts and osteoblasts, respectively. Moreover, the selection of PLGA degradation by-product concentration of 20 mM was selected based on the measured pH of di-H_2_O that was falling around the pH value of 5 along the degradation studies.

It was verified that changing pH values due to different concentrations of PLGA degradation by-products exhibited a dose-dependent effect on oAECs by hindering their viability and proliferation rate, which can negatively influence the regeneration process. Indeed, cells exposed to high non-buffered acidic concentration media underwent a relevant suffering state clearly visible from the changes in cell morphology and the presence of pyknotic nuclei. Additionally, exposing oAECs to degradation media containing LA or GA at concentrations of 5 mM and 10 mM exhibited low toxic effects according to the MTT and cytological results throughout the culture time points. Instead, cells subjected to media containing 20 mM LA or GA showed a reduced viability of around 40% after 24 h of culture accompanied with a drastic decrease in cell number. Cell toxicity was compensated after 48 h of culture, where oAECs were able to proliferate even when exposed to 20 mM of LA- or GA-enriched media. The obtained results can be probably explained by the physiological buffering system that cells possess [28,29,92,93]. On the other hand, after 7 d of culture, when oAECs were exposed to 10 mM of LA or GA, cell viability decreased around 30% and continued to decrease gradually to nearly reach 50% viability at a concentration of 20 mM. Interestingly, oAECs exposed to 40 mM of LA or GA showed a drastic decrease in viability to only 5% at all culture points. The obtained results were in accordance with those published previously by Gil-Castell et al., who demonstrated that exposing fibroblasts to LA or GA exhibited a dose-dependent effect [23].

When cells were subjected to a mixture of acidic media containing both LA and GA (1:1), they remained viable after being cultivated for 24 h and 48 h at low concentrations (5 mM and 10 mM) and only at 5 mM after 7 d culture. Instead, cells lost around 60% of their metabolic activity when subjected to the mixture of acidic by-products at a concentration of 10 mM after 7 d culture. By increasing the concentration of LA:GA in culture (>20 mM), oAECs were unable to proliferate and exhibited a mortality of around 95% at all culture time points. The obtained results demonstrated that the continuous release of PLGA scaffold degradation by-products (LA:GA) possessed a synergic effect on decreasing cell viability. These results were in accordance with those published by Meyer et al., who demonstrated that the exposition of a non-buffered mixture of LA and GA (1:1) to osteoblasts caused a drastic cell death even at a low concentration (15 mM) [91].

Physiologically, the body’s buffer systems are incredibly efficient. In fact, it takes mere seconds for the chemical buffers in the blood to regulate the pH [28]. The maintenance of pH in the face of acid loading is because of ion fluxes across the cell membrane. For example, by expelling CO_2_ from the body, the respiratory tract may raise the blood pH in minutes. Moreover, the renal system may also modify blood pH by excreting hydrogen ions (H^+^) and conserving bicarbonate (HCO_3_^-^), although this process takes hours to days to take effect. The kidneys contribute to acid–base balance by excreting H^+^ and producing HCO_3_^−^, which helps keep blood plasma pH within a normal range [28,29]. The coupled exchange of ions (H^+^ for HCO_3_^−^ and Na^+^ for Cl^−^) is an electroneutral process; this means that the membrane potential remains unaltered [28,29]. The physiological buffer system is regenerated when the concentration of acid loading (H^+^) increases within the body in which the buffer system rapidly neutralizes the excess protons. The net excretion of the protons by the kidney replenishes the base stores, hence regenerating the buffer for further physiological neutralization [28,29].

To this end, the PLGA degradation media were buffered to a physiological pH (7.4) and subjected to oAECs at different concentrations. Interestingly, by using buffered culture media containing the degradation by-products LA, GA, or a mixture of both acids, cells remained viable even at a high concentration (40 mM) after incubation for 24 h and 48 h of culture. These results were in accordance with those obtained by Meyer et al., who demonstrated that by using buffered degradation media composed of both LA and GA at a concentration up to 100 mM, no toxic effect was noted on osteoblasts after 24 h of culture [91]. Instead, at 7 d culture, although the viability of cells subjected to the buffered culture media containing LA or GA remained high at a concentration up to 20 mM, cells’ vitality decreased to reach 60% at a concentration of 40 mM. By using the culture media containing the mixture of both acids, cells’ viability decreased about 40% at a concentration of 10 mM and remained nearly constant up to 40 mM. Cells exposed to the mixture culture media of both LA and GA showed a decreased viability at a concentration of 10 mM after 7 d of culture. It can be noticed that by increasing culture time, cells cultured with buffered media were not able to compensate for the acidic effects of degradation by-products even at low concentrations. The obtained results concerning cytological and MTT analyses confirmed that the exposition of oAECs to a high non-buffered PLGA acidic concentration induced cells’ suffering from the acidic microenvironment.

To date, no studies have explored the effects of acidic degradation by-products of PLGA on modulating oAECs’ gene expression of anti- and pro-inflammatory cytokines. For this reason, the influence of PLGA degradation by-products on oAECs’ immunomodulatory properties became evident by assessing the gene expression profile of anti-(IL-10) and pro-(IL-12) inflammatory cytokines at early (24 h and 48 h) and late (7 d) culture times, mimicking the inflammation state after biomaterial implantation [14,94].

oAECs upregulated the gene expression of IL-10 when subjected to a high concentration (20 mM) of LA, GA, and LA:GA at 24 h and 48 h, while the long-term exposure (7 d) led to an upregulated expression of IL-10 even at low concentrations. Interestingly, when buffered degradation media were used, oAECs expressed lower levels of IL-10 compared to those subjected to non-buffered media. These results could be explained by the fact that oAECs subjected to buffered media, compared to non-buffered ones, were able to survive, maintaining a viability higher than 70% throughout all culture time points, represented by lower cell suffering with lower expression levels of IL-10.

A similar dose-dependent profile was observed by analyzing the gene expression of IL-12 within oAECs when subjected to the different acidic degradation media. When non-buffered acidic media were used, cells upregulated IL-12 expression only when exposed to 20 mM at 7 d culture. Instead, IL-12 was already upregulated within oAECs after 24 h and 48 h when both acidic degradation media were combined, reflecting the synergic effect of both acids on oAECs immunomodulation. However, when buffered media were used, cells exhibited a similar gene expression profile of IL-12 as that obtained for IL-10. To get better insights on these results, the IL-10/IL-12 ratio was assessed according to Manuelpillai et al. [82] and Mauro et al. [95]. Interestingly, oAECs subjected to all degradation culture media exhibited high levels of the IL-10/IL-12 ratio, indicating that oAECs seemed to compensate for the degradation of the PLGA scaffold, enhancing their anti-inflammatory aptitude that could stimulate in vivo the skewing of the macrophage towards the M2 phenotype, thus inducing a rapid transition from inflammatory to reparative phase.

The inflammation process may depend on the degradation rate of the scaffolds, the concentration of the released degradation by-products, and the inflammatory response of damaged tissues. Thus, it is of great interest how released by-products affect the inflammatory response during the degradation of scaffolds to better direct tendon healing towards regeneration and hinder the status of inflammation and hence the rejection of the implantable scaffolds. The obtained results suggested that oAECs that possess immunomodulatory properties can compensate, at the analyzed concentrations, for the effect of released degradation components of PLGA scaffolds by upregulating the gene expression of anti-inflammatory cytokines.

The promising data obtained within this study should be completed according to ISO-10993—part 16 in the future, for the validation of the scaffolds, including the toxicokinetics for degradation products and leachables. Moreover, oAECs should be cultivated with ha-PLGA scaffolds to assess the effect of PLGA degradation by-products on cell performance and the synthesis of neo-formed ECM over a long-time culture. Once everything has been set and verified, the scaffold can be translated to in vivo experimentation for a better validation of the fabricated ha-PLGA construct.

## 5. Conclusions

The biodegradability of polymer is a key factor to consider in designing scaffolds for TE applications. In this study, the degradation profile of electrospun ha-PLGA scaffolds with highly aligned fibers, intended to be applied for tendon regeneration, was assessed under di-H_2_O and PBS conditions for up to 20 weeks. The solid and liquid fractions of the degradable materials were separated and characterized for mass loss; liquid uptake; molecular weight; morphological, ultrastructural, and mechanical property changes; and pH and conductivity changes of the di-H_2_O and PBS media. The obtained results showed that the fabricated ha-PLGA constructs exhibited a slow degradation profile with a degradation half-time of about 40 weeks under di-H_2_O, confirmed by a slight decrease in the mass loss and average molecular weight with a faster kinetic under di-H_2_O compared to PBS conditions that could be related to different factors characterizing the studied material. Moreover, it was demonstrated that the mechanical properties decreased throughout the degradation times, which is considered a critical factor to monitor for tendon regeneration when transplanted in vivo. Considering that the remodeling stage starts 6 to 8 weeks after injury and takes around 1 to 2 years [96], providing a scaffold with adequate intrinsic physical cues and tunable degradation to support tissue damage represents a challenge to be solved. However, a long degradation profile of biodegradable scaffolds could limit complete tissue regeneration, hindering its biomedical applications. On the other hand, the exposure of oAECs to culture media containing PLGA degradation by-products exhibited a dose-dependent effect with a notable effect at the long culture point. The oAECs were able to compensate to a certain level for the created inflammation-like microenvironment by upregulating the gene expression of IL-10, favoring an anti-inflammatory response rather than a pro-inflammatory one. Further studies are still needed to better understand the effect of scaffold degradation and the released degradation by-products on the tenogenic commitment of oAECs, and hence their effects on the production of tendon-like ECM at a long-term culture time. Additionally, further studies on the fabricated ha-PLGA scaffolds are required to improve their wettability, thus tuning their degradation profile by applying non-thermal plasma techniques, such as cold atmospheric plasma, which aim to modify scaffolds’ surface properties and improve cell performance without altering polymer bulk properties.

## Figures and Tables

**Table 1:** pH measurements for each non-buffered acidic medium (LA, GA, and LA:GA) at different concentrations (5, 10, 20, and 40 mM).

Concentration (mM)	pH on Non-Buffered Media		
	LA	GA	LA:GA
5	6.70	6.74	6.32
10	6.34	6.33	5.47
20	5.60	5.40	4.03
40	4.07	4.01	3.46

## Data Availability

The data supporting reported results can be available upon request to the authors.
